# Low Phytate Peas (*Pisum sativum* L.) Improve Iron Status, Gut Microbiome, and Brush Border Membrane Functionality In Vivo (*Gallus gallus*)

**DOI:** 10.3390/nu12092563

**Published:** 2020-08-24

**Authors:** Tom Warkentin, Nikolai Kolba, Elad Tako

**Affiliations:** 1 Crop Development Centre, Department of Plant Sciences, University of Saskatchewan, 51 Campus Dr., Saskatoon, SK S7N 5A8, Canada; tom.warkentin@usask.ca; 2 USDA-ARS, Robert W. Holley Center for Agriculture and Health, Cornell University, Ithaca, NY 14853, USA; nk598@cornell.edu

**Keywords:** pea, phytate, iron, bioavailability, bio active compound, in vivo, *Gallus gallus*, brush border membrane, microbiome

## Abstract

The inclusion of pulses in traditional wheat-based food products is increasing as the food industry and consumers are recognizing the nutritional benefits due to the high protein, antioxidant activity, and good source of dietary fiber of pulses. Iron deficiency is a significant global health challenge, affecting approximately 30% of the world’s population. Dietary iron deficiency is the foremost cause of anemia, a condition that harms cognitive development and increases maternal and infant mortality. This study intended to demonstrate the potential efficacy of low-phytate biofortified pea varieties on dietary iron (Fe) bioavailability, as well as on intestinal microbiome, energetic status, and brush border membrane (BBM) functionality in vivo (*Gallus gallus*). We hypothesized that the low-phytate biofortified peas would significantly improve Fe bioavailability, BBM functionality, and the prevalence of beneficial bacterial populations. A six-week efficacy feeding (*n* = 12) was conducted to compare four low-phytate biofortified pea diets with control pea diet (CDC Bronco), as well as a no-pea diet. During the feeding trial, hemoglobin (Hb), body-Hb Fe, feed intake, and body weight were monitored. Upon the completion of the study, hepatic Fe and ferritin, pectoral glycogen, duodenal gene expression, and cecum bacterial population analyses were conducted. The results indicated that certain low-phytate pea varieties provided greater Fe bioavailability and moderately improved Fe status, while they also had significant effects on gut microbiota and duodenal brush border membrane functionality. Our findings provide further evidence that the low-phytate pea varieties appear to improve Fe physiological status and gut microbiota in vivo, and they highlight the likelihood that this strategy can further improve the efficacy and safety of the crop biofortification and mineral bioavailability approach.

## 1. Introduction

Micronutrient malnutrition affects more than half of the global population, primarily in developing regions [1,2]. Iron (Fe), zinc (Zn), and vitamin A deficiencies are prominent health constraints worldwide [3]. In low-income countries, plants are the significant source of food. In crude cereal and legume foods, the low bioavailability of Fe and Zn leads to metabolic disorders that are associated with these nutritional factors. Hence, increasing the nutritional value of such types of dietary ingredients will contribute to the nutritional status of the target population. Mineral, phosphorous, and phytate content is much higher in bran than whole grain [4,5,6].

Field pea (*Pisum sativum* L.) is a main pulse crop grown for human consumption as a source of protein, carbohydrates, minerals, and bioactive plant-origin bioactive compounds, contributing to better metabolic health. In 2014, the global production of peas was 11.2 million tons [7]. The main component of pea is starch, which includes two polymers of d-glucose: amylose and amylopectin [8,9]. Because of the alterations in physiochemical characteristics between pulses and cereal starches, starch from pulses can deliver some specific features to food systems as high gelation temperature, resistance to shear thinning, increased elasticity, and high concentration of resistant starch [10].

In addition, field peas include bioactive compounds such as oligosaccharides, polyphenols, and phytate [11]. Water-soluble carbohydrates in peas comprise mostly disaccharides and oligosaccharides. The raffinose group of oligosaccharides (RFOs) is the most targeted in pea research. These factors include galactose molecules (linked by α-d-1, 6-glycosidic bonds) attached to sucrose [12]. Humans lack the essential enzymes that are essential to break down these RFOs, and this results in these oligosaccharides being digested by intestinal bacterial populations via fermentation, leading to elevated short-chain fatty acid production [13]. Furthermore, a recent study indicated that intra-amniotic administration of raffinose upregulated the expression of brush border membrane (BBM) functional proteins, downregulated the expression of Fe-related proteins (indicating improvement of dietary iron bioavailability), and elevated villus surface area. Furthermore, raffinose increased the richness and composition of probiotic populations, and it reduced that of pathogenic bacterial species. Overall, raffinose improved microbial population, dietary Fe bioavailability, and BBM functionality in vivo [14].

The main phenolic compounds found in peas comprise condensed tannins, flavonoids, and phenolic acids [15]. These phenolic compounds are found specifically in the seed coat and are biosynthesized via the phenylpropanoid pathway, with condensed tannin molecules being responsible for the seed-coat coloring [16]. In dark-colored hulls, tannin and flavonoid compounds are the majority of phenolic compounds; however, in seeds with clear hulls, phenolic acids are the main compounds [17]. Polyphenols in the seed coat present antioxidant and anti-mutagenic activity, shielding the seed from oxidative stress [18]. In field conditions, these compounds also deliver chemical resistance against pathogens and insect pests during the growing process of the plant [19]. Polyphenols in peas appear mostly as insoluble or bound forms, covalently bonded to structural components of the cell wall such as cellulose, hemicellulose, lignin, and pectin [20,21]. The polyphenolic composition of peas is predominantly interesting with respect to metabolic health, given their alleged protective properties against oxidative stress [15,22]. According to Campos-Vega [11] and Rochfort [23], isoflavone polyphenols are linked with biological pathways in the lessening of osteoporosis and cardiovascular disease, the deterrence of cancer, and treating symptoms related to menopause. Phenolic compounds also display anti-nutritional effects, and related research showed a decrease in the bioavailability of proteins triggered by phenolic compounds [24]. Phytate functions as a storage for phosphate and minerals in seeds that can be recovered during germination process [25]. Phytate was recognized as an anti-nutrient due to its ability to chelate with multivalent ions, specifically Zn, Ca, and Fe, inhibiting the body’s capability to absorb dietary minerals by limiting their bioavailability [24]. There is increasing interest in utilizing pulses in wheat-based products with blends [26]. The demand for gluten-free products led to investigation of the nutritional characteristics of baked products from pulses like chickpea and lentil [27], as well as peas [28]. The rheological properties of pea flour, including the gelation properties of starch, may be considered when exploring the potential application of pea flour in baked goods. Recent uses for pulses could increase the demand for pulses with specific nutritional and rheological properties, which will increase the need to investigate the components affecting the nutritional and functional properties of pulses. It was previously demonstrated that low-phytate pea lines had higher Fe bioavailability than regular or standard pea [29]; in addition, pea varieties which were low-phytate combined with relatively higher carotenoid concentration in some cases resulted in a further increase in Fe bioavailability in vitro [30].

Biofortified staple foods are an effective instrument through which to address micronutrient deficiencies worldwide, with emphasis on Fe and Zn, in numerous target populations [1,31,32,33,34,35]. The in vivo (*Gallus gallus*) model was established as an excellent model to assess dietary Fe and Zn bioavailability [33,34,35,36,37,38,39]. Hence, the objective of the current study was to evaluate the ability of low-phytate pea varieties in the context of a complete meal to improve Fe bioavailability and absorption, physiological status, intestinal BBM functionality, and intestinal microbial populations in vivo (*Gallus gallus*). We suggest the further use of in vivo screening model to guide future studies aimed to investigate biofortified staple food crops, as this method will allow proceeding to human efficacy studies with superior confidence and success.

## 2. Materials and Methods

### 2.1. Plants Materials—University of Saskatchewan Pea Varieties

**Figure 1:**
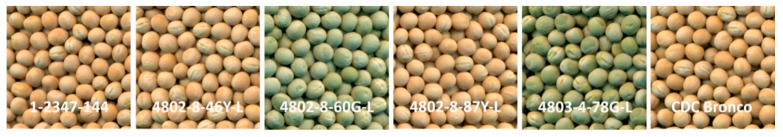
High-resolution photographs depicting six varieties used to evaluate the iron bioavailability of the Saskatchewan peas. To compare the differences in seed sizes, all photographs were taken to scale under standardized lighting conditions.

The pea varieties evaluated in this research arose from the Crop Development Center, University of Saskatchewan (Canada) pea breeding program (Figure 1). Low-phytate line 1-2347-144 was derived from cultivar CDC Bronco [39,40] through chemical mutagenesis [41]. Varieties 4802-8-46Y-L, 4802-8-60G-L, and 4802-8-87Y-L resulted from the cross 1-2347-144/CDC 2235-4 made in 2011. CDC 2235-4 was later registered as CDC Raezer [42]. Variety 4803-4-78G-L resulted from the cross 1-150-81/CDC 2336-1 made in 2011. Line 1-150-81 is a second low-phytate line derived from CDC Bronco [41]. CDC 2336-1 was later registered as CDC Limerick [43]. The varieties from crosses 4802 and 4803 were previously described [30].

### 2.2. Growing Conditions and Post-Harvest Handling

All six pea varieties that were used in this experiment were grown at the Sutherland farm, located 10 km east of Saskatoon (Canada), with planting in May 2017 and harvest in August 2017. The harvested samples were stored in a non-heated warehouse, with temperature ranging between 15 and 20 °C based on the season, until shipment to Ithaca for dietary processing.

### 2.3. Ingredient Preparation and Diet Composition

For this study, raw pea seeds were rinsed and cleaned thoroughly in distilled water to remove dust, debris, and non-edible material. Peas were pre-soaked in distilled water (1:6 *w*/*w*) for 12 h at room temperature prior to cooking. Peas were cooked in boiling distilled water in stainless-steel steam kettles. Cooked peas were then stored at −20 °C for 24 h prior to freeze-drying (VirTis Research Equipment, Gardiner, NY, USA). Basmati rice and wheat were purchased from a local food store located in Ithaca, New York, USA. Our rationale with regard to the inclusion of basmati rice, wheat, and carrots in the tested pea-based diets was to approximately simulate the ingredients of a pea-based meal in India, which is one of the key consumers of pea, and where dietary Fe deficiency is a major health concern. Cooked rice was stored at −20 °C for 24 h before freeze-drying. Cooked/air-dried carrots were purchased from North Bay Trading Co. (Brule, WI, USA). Dried ingredients were milled into a course powder using a Waring Commercial^®^ CB15 stainless-steel blender (Torrington, CT, USA). Other dietary ingredients included chicken Vitamin Mixture (#330002) and chicken Mineral Mix (#230000, no added iron) (Dyets Inc., Bethlehem, PA, USA), dl-methionine, and choline chloride (Sigma-Aldrich, St. Louis, MO, USA). The compositions of the experimental diets are shown in Table 1

### 2.4. Iron Analysis

Iron analysis was conducted as previously described [14,33,36,38,39]. For the analysis, a 500-mg sample of dietary ingredient, a 500-mg sample of pea-based diets, or a 100-mg sample of tissue (wet weight) was analyzed.

### 2.5. Phytate Analysis

Phytate (phytic acid) determination was conducted as previously described [14,33,34,35,36,37,38,39]. For the analysis, a 500-mg sample of dietary ingredients and a 500-mg of pea-based diets were analyzed, according to a phosphorous kit (K-PHYT; Megazyme International, Ireland).

### 2.6. Protein and Fiber Analysis

Analysis was conducted as previously described [36,43,44,45].

### 2.7. Animals and Feeding Trial Design

Cornish-cross fertile broiler eggs were delivered from a commercial hatchery (Moyer’s Chicks, Quakertown, PA, USA). The eggs were incubated under ideal conditions at the Cornell University Animal Science poultry farm incubator. Upon hatch (hatchability = 98%), hatchlings were arbitrarily divided into seven treatment groups (*n* = 15) (Table 1), with ad libitum access to food and water (Fe concentration < 0.4 μg/L). Chicks were kept in a total confinement building (two animals per 1-m^2^ metal cage) under controlled temperature and humidity with 16 h of light. Cages were equipped with an automatic watering system and a manual self-feeder. Feed intakes were documented daily, and, as of day of hatch, body weights were documented weekly. Animal protocols were approved by the Cornell University Institutional Animal Care and Use Committee (protocol number 2007-0129).

#### 2.7.1. Blood Collection, Hemoglobin, and Physiological Fe Status Parameters

Blood samples were collected and hemoglobin (Hb) assays were conducted according to the Hb kit manufacturer’s instructions (BioAssay Systems, Hayward, CA, USA). Total body hemoglobin Fe (Hb-Fe), a parameter of iron absorption, was calculated from Hb concentrations and blood volume according to specific body weight (85 mL per kg of body weight) [33,34,35,36,39,46]. 

Hemoglobin maintenance efficiency (HME) was calculated as the cumulative difference in total body Hb Fe from the start of the study, divided by total dietary Fe intake. [33,34,35,36,39,46].

Upon the conclusion of the study (42 days), animals were euthanized by CO_2_ exposure and blood, small intestine, cecum, and liver samples were collected. Tissue samples were instantly frozen in liquid nitrogen and stored at −80 °C in a freezer until analyzed.

#### 2.7.2. Liver Iron and Ferritin

The quantifications of liver Fe and ferritin were conducted as previously described [46,47,48].

#### 2.7.3. Isolation of Total RNA from Duodenum

Total RNA extraction was conducted as previously described [14,33,34,35,36,37,38,39,46,49], according to the manufacturer’s protocol (RNeasy Mini Kit, Qiagen Inc., Valencia, CA, USA).

#### 2.7.4. Real-Time Polymerase Chain Reaction (RT-PCR)

The complementary DNA (cDNA) reaction was conducted as previously described (BioRad C1000 touch thermocycler using the Improm-II Reverse Transcriptase Kit, Promega Corp., Madison, WI, USA) [37,38,39].

#### 2.7.5. Primer Design for Duodenal Gene Expression

Primers sequences were designed and selected using the Real-Time Primer Design Tool software (IDT DNA, Coralvilla, IA, USA). The *Gallus gallus* primers (forward/reverse) that were used in this study are indicated in Table 2.

#### 2.7.6. Real-Time qPCR Design

Isolated cDNA was used for the reaction (Cat. #1725274, Hercules, CA, USA) as previously indicated [36,37,38,39].

#### 2.7.7. Collection of Microbial Samples and DNA Isolation of Intestinal Contents

The cecum was removed and stored at −80°C until analyzed. Microbial DNA isolation was conducted as previously described [36,37,38].

#### 2.7.8. Primer Design and PCR Amplification of Bacterial 16S rRNA

Primers for *Bifidobacterium, Lactobacillus, Escherichia coli*, and *Clostridium* were used in accordance with previously published data [46].

#### 2.7.9. Glycogen Analysis

At the conclusion of the study (day 42), the pectoral muscle (200 mg) was removed, and glycogen contents were determined as previously described [50,51,52].

### 2.8. Statistical Analysis

Statistical analyses were conducted using IBM SPSS Statistics 25 (IBM Analytics, Armonk, NY, USA). Measured parameters were found to have a normal distribution and equal variance, and they were acceptable for ANOVA. Mean separations for measured parameters were determined using ANOVA with the model including dietary treatment (seven levels) as the fixed effect, followed by a Duncan post hoc test. Differences with *p*-values ≤0.05 were considered statistically significant.

## 3. Results

### 3.1. Seed Iron and Phytate Concentrations in Experimental Peas Varieties

Iron concentrations of dietary ingredients are shown in Table 1. Differences in seed Fe contents in the pea varieties were significant (*p* ≤ 0.05), ranging from 37 μg/g in 1-2347-144 to 42 μg/g in 4803-4-78G-L (Table 1). Phytate concentrations and molar ratios of dietary ingredients of the pea-based diets are indicated in Table 1. Significant (*p* ≤ 0.05) differences in phytate concentrations were measured between peas varieties, from 3.7 mg/g in 4803-4-78G-L to 5.82 mg/g in CDC Bronco (Table 1). Phytate-to-Fe molar ratios varied significantly (*p* ≤ 0.05), from a ratio of 7.4 in 4803-4-78G-L to a ratio of 12.4 in CDC Bronco (Table 1).

### 3.2. Protein and Fiber Contents

Table 3 indicates the total crude protein content in experimental tested pea varieties, with significant differences (*p* ≤ 0.05) between pea varieties, ranging from 22.5 g/100 g in CDC Bronco to 26.75 g/100 g in 4803-4-78G-L. Concentrations of insoluble, soluble, and total fiber for experimental peas are shown in Table 3, with significant differences (*p* ≤ 0.05) in each of the fiber fractions between experimental peas. The lowest concentrations of the insoluble, soluble, and total fiber were detected in the 4803-4-78G-L pea variety. Significantly (*p* ≤ 0.05) higher concentrations of all three fiber fractions were measured in 1-2347-144. As a reference, the total protein content in the control diet (no pea) was measured at 10.72 g/100 g ± 0.16 g/100 g of total protein.

### 3.3. Iron–Phytate Analysis of Pea Based Diets

The final composition of the six pea-based diets and no-pea diet are shown in Table 3. Iron concentrations amongst the pea-based diets were significantly different (*p* ≤ 0.05). Diets formulated from 4802-8-87Y-L and 4803-4-78G-L had the highest iron concentrations (38 μg/g and 39 μg/g, respectively) relative to the control diet (no-pea diet) (27 μg/g). Final phytate concentrations also varied between experimental diets ranging from 1.57 mg/g in 1-2347-144 to 2.66 mg/g in the no-pea diet. Significant (*p* ≤ 0.05) differences in phytate–Fe molar ratios were observed between the pea-based diets, ranging from 3.79 mg/g in 1-2347-144 to 8.66 mg/g in CDC Bronco (Table 1).

### 3.4. In Vivo Assay (Gallus gallus Feeding Trial)

#### 3.4.1. Growth Rates, Hemoglobin (Hb), Total Body Hemoglobin Fe (Hb-Fe), and Hemoglobin Maintenance Efficiency (HME)

Feed intakes and Fe intakes were higher (*p* < 0.05) in all pea-based dietary treatment groups relative to the no-pea dietary treatment group (Table 4 and Table 5).

Also, as from day 35 of the study, body weights were consistently higher (*p* < 0.05) in several of the low phytate pea based dietary groups (4803-4-78G-L, and 4802-8-87Y-L), relative to the CDC Bronco and no-pea dietary groups (Table 6). Hemoglobin (Hb) values did not differ between treatment groups; however, significant differences in total body Hb-Fe, a physiological biomarker of Fe bioavailability and status, were detected as of week five of the study (Table 7), demonstrating an improvement in Fe status in the 4802-8-87Y-L group, relative to CDC Bronco and no-pea diet groups. In addition, the standard pea variety treatment group (CDC Bronco) had a lower HME (*p* < 0.05) at each time point when compared to the group receiving the lower-phytate pea-based diets (groups 1-2347-144, 4803-4-78G-L), indicating a higher dietary Fe bioavailability and increased absorbable Fe (Table 8).

#### 3.4.2. Hepatic Iron and Ferritin Concentrations

The contents of liver iron and ferritin (day 42) are shown in Table 9. Significant (*p* ≤ 0.05) differences in liver iron were detected among the seven treatment groups with concentrations ranging from 73 μg/g in the group receiving the 4803-4-78G-L diet to 96 μg/g in the 1-2347-144 diet. Significant (*p* ≤ 0.05) differences in liver ferritin concentrations were also measured between the seven dietary treatment groups (Table 9).

#### 3.4.3. Serum Iron Concentrations

Significant differences (*p* ≤ 0.05) in serum iron concentrations were detected on day 21 and 35 of the study. On day 21, the lowest concentration of serum iron was 1.526 µg/µL in the no-pea dietary group, while the highest concentration was in the 4802-8-87Y-L pea-based dietary group (2.812 µg/µL). On day 35, the lowest concentration of serum iron was 1.488 µg/µL (no-pea dietary group), while the highest concentration was detected in the 4803-4-78G-L dietary group (2.633 µg/µL) (Table 10).

#### 3.4.4. Glycogen Concentrations in Pectoral Muscle

As an indicator of energetic status [52,53], pectoral muscle glycogen concentrations were measured on days 21 and 42 of the study (Table 11). No significant differences were detected on day 21; however, significant differences (*p* ≤ 0.05) were measured on day 42 in the abundance of glycogen stored in pectoral muscles. The highest values of glycogen were in the 4802-8-60G-L pea-based dietary group, and the lowest concentration of glycogen was in the no-pea dietary group.

#### 3.4.5. Duodenal Gene Expression

**Figure 2:**
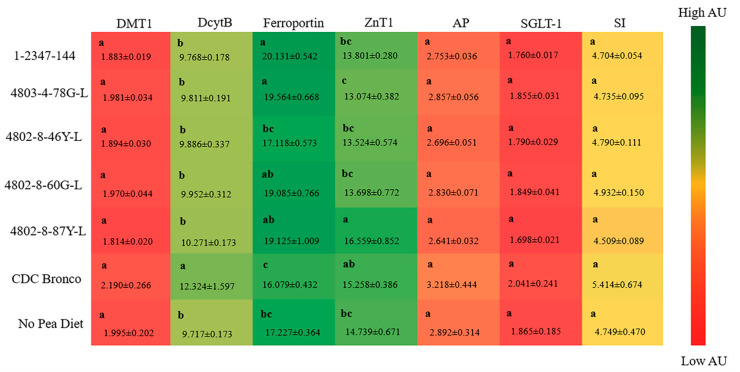
Gene expression of iron proteins in the duodenum after six weeks of consuming pea-based diets. Values are means ± SEM (*n* = 10 per treatment group). ^a–c^ Treatment groups not indicated by the same letter are significantly different (*p* < 0.05). DMT-1, divalent metal transporter-1; DcytB, duodenal cytochrome b; ZnT1, zinc transporter 1; AP, amino peptidase; SGLT-1, sodium-glucose transporter 1; SI, sucrose isomaltase.

The duodenal gene expression of iron- and zinc-related proteins, as well as BBM functional proteins, is shown in Figure 2. Significant (*p* ≤ 0.05) differences in the expression of DcytB and ferroportin were identified, with no significant differences in divalent metal transporter-1 (DMT1) expression between treatment groups.

#### 3.4.6. Cecum Content Bacterial Populations Analysis

**Figure 3:**
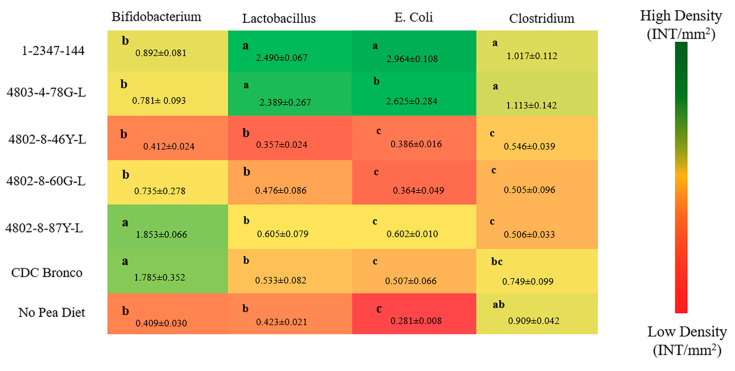
Genus- and species-level bacterial populations (AU) from cecal contents after six weeks of consuming pea-based diets. Values are means ± SEM (*n* = 10 per treatment group). ^a–c^ Treatment groups not indicated by the same letter are significantly different (*p* < 0.05).

As shown in Figure 3, the relative abundance of *Bifidobacterium* was significantly higher (*p* < 0.05) in the 4802-8-87Y-L and CDC Bronco groups relative to all other treatment groups. Furthermore, the abundance of *Lactobacillus* was significantly higher (*p* < 0.05) in the 1-2347-144 and 4803-4-78G-L groups relative to all other treatment groups.

## 4. Discussion

The objective of the current study was to investigate the effects of low-phytate peas, in the context of a complete meal, on Fe bioavailability, absorption, physiological status, intestinal BBM functionality, and gastrointestinal microbial populations in vivo (*Gallus gallus*).

In studies of biofortification, the process via which the nutritional quality of food crops is improved through agronomic practices, conventional plant breeding, or modern biotechnology [2], it is necessary and advantageous to utilize in vivo screening tools that are capable of assessing biofortified varieties of staple crops, as well as in relation to the diet in which they are consumed [1,33,36,38,39,46,54,55,56]. The present study, for the first time, presents a demonstration of how the *Gallus gallus* model of Fe (and Zn) bioavailability could be useful in the design of the current study aimed at assessing the potential nutritional benefit of lower-phytate versus standard peas. The chosen dietary composition was specifically formulated in accordance to a potential target population (Indian/Bangladeshi pea-based dal meal), similar to previous in vivo studies aimed at assessing dietary Fe bioavailability in beans [35,55] and wheat [38] (Table 1). Overall, our data agree with previously published knowledge [1,39,49,55], demonstrating that this in vivo screening approach is effective in the evaluation process of nutritional qualities of the low-phytate pea varieties. Furthermore, the data suggested that lower-phytate pea-based diets were able to moderately improve Fe physiological status in vivo.

Peas are a common staple food crop consumed worldwide, primarily in India, China, Russia, Ethiopia, and Bangladesh. Global dry pea production increased from 9.9 million tons in 2012 to 16.2 million tons in 2017 [7]. Currently, the leading producers are Canada, Russia, China, Ukraine, and India. In Canada, a leading producer and exporter of dry peas, pea was grown on 1.6 million ha in western Canada (Saskatchewan, Alberta, and Manitoba) in 2017, indicating a significant alteration in cropping practices from the 300 ha reported in 1967. Pea was the major alternative crop as farmers shifted toward a more diversified crop production. Pea varieties (yellow and green cotyledon) are grown, with an average of 80% production in yellow cotyledon varieties. The five-year (2013–2017) average pea yield in western Canada is 2.6 tons/ha (38 bu/ac) [57]. As for their nutritional value, it was previously demonstrated that pea seeds are high in protein, carbohydrates, fiber, B vitamins, and minerals (potassium, magnesium, calcium, iron), and they are considered an inexpensive source of energy-dense, nutrient-rich food [58,59,60]. In addition, pea seeds are low in fat and cholesterol-free. Because of these nutritional benefits, worldwide pea utilization is expected to continue to grow.

Plant seeds, such as pea, contain a high concentration of phosphorus. However, about 60–80% of the total phosphorus in seeds is stored in the form of phytate, a mixed-cation salt of phytic acid [59]. This introduces a nutritional challenge, as negatively charged sites of phytic acid bind and form salts with K^+^, Mg^2+^, Ca^2+^, Mn^2+^, Zn^2+^, or Fe^3+^ [61]. Phytate causes multiple difficulties, as non-ruminant animals including pig, poultry, fish, and humans, are unable to digest phytate due to lack of a phytase enzyme [61]; as a result, important micronutrients (as Zn^2+^ and Fe^3+^) bound to phytate are also excreted and not absorbed, potentially leading to micronutrient deficiencies [62]. Recently, the development of cultivars with low-phytate content became an effective approach to potentially reducing nutritional concerns ascending from the consumption of phytate-rich grains. Low-phytate varieties were chemically persuaded in maize (*Zea mays* L.) [63], soybean (*Glycine max* (L.) Merr.) [64], barley (*Hordeum vulgare* L.) [63,65], rice (*Oryza sativa* L.) [66], wheat (*Triticum aestivum* L.) [67], bean (*Phaseolus vulgaris* L.) [68], and pea [41]. The concentration of phytate phosphorus is significantly reduced in the mutants with an associated increase in available phosphorus. Wilcox et al. [65] reported an 80% reduction in phytate phosphorus content in a low-phytate soybean mutant, as compared with its nonmutant sibling, and this reduction was matched by an equal increase in inorganic phosphorus.

It was previously demonstrated that low-phytate crops increase the bioavailability of phosphorus and several important nutritional cations, including Fe. These crops could assist in increasing the health of a large proportion of the global population, which is dietary Fe-deficient, primarily in target regions where dietary peas are consumed regularly. For example, in a previous study focused on the nutritional evaluation of low-phytate pea diets in vivo, it was demonstrated that animals fed the low-phosphorus diets had lower weight gain and feed intake (*p* < 0.01) than those fed the higher phosphorus level. Bone strength was higher (*p* < 0.01) for animals fed diets based on low-phytate pea than for those fed diets based on normal pea or soybean meal. The authors concluded that increasing the availability of the phosphorus in peas could mean that less inorganic phosphorus would be required in order to meet the nutritional requirements of broilers [59].

In the context of the current study, the results indicated that, despite Hb levels not being significantly higher in the lower-phytate pea groups, significant differences in total body Hb-Fe, the physiological Fe status biomarker [33,34,35,36,39,46,55], were observed (Table 8), representing an enhancement in Fe status in the 4802-8-87Y-L dietary group, relative to CDC Bronco and the no-pea dietary group. In addition, the standard pea variety (CDC Bronco) treatment group had a lower HME (*p* < 0.05) ratio compared to the group receiving the lower-phytate pea-based diets (groups 1-2347-144, 4803-4-78G-L) (Table 8), indicating improved dietary Fe bioavailability and increased absorbable Fe [36,46,54]. The CDC Bronco diet presented a higher PA–Fe ratio compared to the all low-phytate pea-based diets (Table 1), which was associated with increased dietary Fe bioavailability in these pea-based diets [69,70,71]. These results agree with preceding experiments intended to assess Fe bioavailability in Fe-biofortified legumes, such as black beans [72], red mottled beans [33], Carioca beans [36], and pearl millet [73], as well as in the context of a complete diet. Thus, several intrinsic factors, including phytates, may influence the bioavailability of Fe from these pea varieties and other crops [56,74,75,76], potentially limiting their nutritional benefit.

Previous research suggested that increased Fe content alone in biofortified crops may not be adequate to produce a significant physiological improvement in Fe status and in Fe-deficient populations [36,55,76]. In the current study, it appears that, although Fe contents of all tested pea varieties were similar, the consumption of lower-phytate peas was able to moderately improve Fe status and storage, as further suggested by the hepatic ferritin contents of lower-phytate groups relative to CDC Bronco and no-pea diets. Furthermore, the duodenal brush border membrane (BBM) gene expression of ferroportin (FPN) was significantly upregulated, while DcytB was downregulated in the groups receiving the lower-phytate pea-based diets, relative to the CDC Bronco dietary group (*p* < 0.05, Figure 2). However, no significant alterations in the expression of BBM functional proteins were detected amongst treatment groups. Previous studies showed a downregulation of the gene expression of Fe-related BBM proteins (DMT-1, FPN, and Dcytb) in Fe-biofortified diets compared to the Fe-standard diets [36,46,55]. Ferroportin is the Fe exporter that transfers Fe across the enterocyte’s basolateral membrane [77]. Hence, since the lower-phytate pea-based dietary groups had a higher expression of FPN, more Fe could be transported from the enterocyte into the blood and target tissue; therefore, this mechanism indicates the potential increased amount of absorbable Fe and, hence, the total body Hb-Fe increased in some of the low phytate groups compared to the CDC Bronco and no-pea dietary groups.

Similar to humans and most animals, the *Gallus gallus* model harbors a complex and active intestinal microbiota [78], significantly and directly influenced by host genetics, environment, and diet [79]. There is a significant resemblance at the phylum level between the gut microbiota of *Gallus gallus* and humans, with Bacteroidetes, Firmicutes, Proteobacteria, and Actinobacteria representing the dominant bacterial phyla in both [80]. In this study, a genus- and species-level bacterial population delineation among the low-phytate, standard (CDC Bronco), and no-pea dietary groups was observed. Results indicated that the abundance of *Bifidobacterium* was significantly higher (*p* < 0.05) in the 4802-8-87Y-L and CDC Bronco groups relative to all other treatment groups. Furthermore, the abundance of *Lactobacillus* was significantly higher (*p* < 0.05) in the 1-2347-144 and 4803-4-78G-L treatment groups relative to all other treatment groups (Figure 3). These results suggest that the above lower-phytate pea-based diets may potentially improve the host overall gut health by promoting the abundance of beneficial bacterial populations. Moreover, some of the low-phytate pea varieties (as 1-2347-144) presented a higher (*p* < 0.05) total fiber content (soluble and insoluble) compared to the standard CDC Bronco pea (Table 3). It was previously demonstrated that soluble fiber can increase villi height by elevating intestinal cell proliferation [81]. In the current study, some of the low-phytate pea dietary groups (such as 4803-4-78G-L, 4802-8-46Y-L, and 4802-8-87Y-L) presented higher (*p* < 0.05) protein content compared to the standard CDC Bronco pea (Table 3), where a higher dietary protein content was shown to increase villi height and intestinal cell proliferation [82]. Furthermore, indigested dietary proteins and fibers are fermented in the lower intestine, and this action produces short-chain fatty acids (SCFAs), such as acetate, propionate, and butyrate. Production of SCFAs affects metabolism and gastrointestinal health [83]. Acetate and propionate are energy substrates for peripheral tissues, and butyrate is referentially used as an energy source by colonocytes [84,85].

In summary, the current study focused on the performance of low-phytate pea varieties in chicken diets. Phytate phosphorus concentration was reduced by approximately 40% in these varieties. The low-phytate pea variety-based diets were able to moderately improve the Fe status in vivo, suggesting that low-phytate field pea has the potential to improve Fe bioavailability in human diets, particularly in the Indian subcontinent, as one of the major importing regions for Canadian peas, and a region where dietary Fe deficiency is a major health concern. Furthermore, as the abolition of micronutrient malnutrition remains a widespread global health problem in developing countries, the current study suggests that increasing micronutrient intake in food through food-based approaches is a sustainable method for the potential prevention of micronutrient deficiencies. Biofortification offers a long-term, sustainable, food-based solution for a world population, and breeding programs may aim to improve grain Zn and Fe concentrations; however, as previously suggested, improving Fe or Zn content may not necessarily result in the desired outcome (i.e., breeding toward increased mineral content may also lead to increased potential dietary inhibitors) and, hence, may not be as effective. In low-income countries, breeding for mineral solidity may remain the only agricultural involvement available to improve the nutritional content of staple crops, and, as suggested in the current study, the genetic improvement of staple food crops, specifically the development of low-phytate pea verities, resulted in improved nutritional quality and dietary Fe bioavailability, including in a complete diet context.

Additionally, as previously demonstrated, the current study presents a cost-effective approach designed to assess the effectiveness of biofortified pea varieties in vivo, as these varieties were developed with an aim to reduce the inhibitory effect of dietary phytate on Fe bioavailability. Therefore, our findings suggest that the use of lower-phytate biofortified peas may be an effective and sustainable approach to decreasing the global abundance of Fe deficiency, with added improvements in intestinal bacterial population structure and intestinal BBM functionality.

## 5. Conclusions

Nutritional approaches aimed to ease global Fe deficiency, such as Fe supplementation or fortification, are moderately successful at achieving optimal Fe status. This study showed how biofortified low-phytate pea affects dietary Fe bioavailability, physiological status, and the composition and metagenome of the gut microbiota and intestinal function. Animals (*Gallus gallus*) that consumed the low-phytate pea-based diets had increased abundance of beneficial bacteria, with associated surges in SCFA-producing bacteria with known phenolic catabolic capability, which resulted in an improvement in intestinal functionality. In addition, some of the low-phytate peas presented a higher protein content versus the standard CDC Bronco pea, which can possibly improve Fe bioavailability and intestinal functionality. Furthermore, parallel to preceding data, the current research suggests that a key aspect to include is the in vivo measurement of dietary Fe bioavailability in biofortified crop variety-based diets, as part of the plant breeding procedure.

Overall, our discoveries provide further evidence that, unlike other nutritional approaches to improving Fe status, the low-phytate pea varieties appear to improve Fe physiological status and gut microbiota in vivo, and they present an option for this strategy to further advance the efficacy and safety of crop biofortification and mineral bioavailability. We recommend the application of in vivo screening tools to guide studies aimed at developing and appraising Fe bioavailability in biofortified food crops, as well as their possible nutritional benefit. Based on the data presented in the current study, a human efficacy study will be conducted to compare the 4802-8-87YL (low phytate) and CDC Bronco (standard/normal phytate) varieties, along with a no-pea control.

## Figures and Tables

**Table 1:** Composition of the experimental pea-based diets ^1^.

	Iron	Dietary Formulation (g/kg)						
Ingredient ^1^	(μg/g) ^2^	1-2347-144	4803-4-78G-L	4802-8-46Y-L	4802-8-60G-L	4802-8-87Y-L	CDC Bronco	No Pea
1-2347-144	37.687 ± 0.106 ^a^	500	−	−	−	−	−	−
4803-4-78G-L	42.512 ± 0.388 ^b^	−	500	−	−	−	−	−
4802-8-46Y-L	41.277 ± 0.258 ^c^	−	−	500	−	−	−	−
4802-8-60G-L	38.020 ± 0.275 ^d^	−	−	−	500	−	−	−
4802-8-87Y-L	39.539 ± 0.285 ^d^	−	−	−	−	500	−	−
CDC Bronco	39.850 ± 0.283 ^d^	−	−	−	−	−	500	−
No pea	−	−	−	−	−	−	−	−
Wheat (whole)	43.863 ± 0.320	150	150	150	150	150	150	400
Basmati rice	4.367 ± 0.028	150	150	150	150	150	150	400
Carrots	25.717 ± 4.762	50	50	50	50	50	50	50
Milk powder	1.742 ± 0.103	50	50	50	50	50	50	50
Vitamin/mineral premix ^3^	0.00 ± 0.0	70	70	70	70	70	70	70
Oil	0.00 ± 0.0	30	30	30	30	30	30	30
DL-Methionine	0.00 ± 0.0	2.5	2.5	2.5	2.5	2.5	2.5	2.5
Choline chloride	0.00 ± 0.0	0.75	0.75	0.75	0.75	0.75	0.75	0.75
Total composition (g)		1000	1000	1000	1000	1000	1000	1000
Pea Only Analysis ^4^								
Phytate concentration (mg/g)		3.96 ± 0.04 ^d^	3.74 ± 0.05 ^e^	4.38 ± 0.01 ^c^	4.76 ± 0.04 ^b^	3.84 ± 0.05 ^de^	5.82 ± 0.01 ^a^	−
Phytate–iron molar ratio		8.90 ^c^	7.44 ^e^	8.98 ^c^	10.59 ^b^	8.21 ^d^	12.36 ^a^	−
Dietary Analysis ^4^								
Iron concentration (μg/g)		35.717 ± 0.378 ^b^	39.473 ± 1.089 ^a^	36.017 ± 0.370 ^b^	36.274 ± 0.302 ^b^	38.087 ± 0.448 ^ab^	37.208 ± 0.157 ^ab^	27.603 ± 1.754 ^c^
Phytate concentration (mg/g)		1.57 ± 0.01 ^e^	2.30 ± 0.32 ^c^	2.62 ± 0.04 ^c^	2.34 ± 0.32 ^c^	3.03 ± 0.10 ^b^	3.32 ± 0.08 ^a^	1.88 ± 0.14 ^d^
Phytate–iron molar ratio		3.71 ^d^	4.93 ^cd^	5.52 ^bc^	5.46 ^bc^	6.73 ^ab^	7.55 ^a^	5.75 ^bc^

^1^ Food constituents were cooked, drained, and lyophilized before milling and for chemical analysis. ^2^ Values are means ± SEM (*n* = 5). ^3^ Vitamin and mineral premix: #330,002 Chick vitamin mixture; #230,000 Salt mix (no iron) for chick diet (Dyets Inc., Bethlehem, PA, USA). ^4^ Values are means ± SEM of five replicates for each of the pea-based diets. ^a–e^ Treatment groups not indicated by the same letter are significantly different (*p* ≤ 0.05).

**Table 2:** Sequences of primers used in this study.

Gene ^1^	Forward Primer (5′–3′)	Reverse Primer (5′–3′)	Length (bp)	GI ID
DMT-1	TTGATTCAGAGCCTCCCATTAG	GCGAGGAGTAGGCTTGTATTT	101	206597489
Ferroportin	CTCAGCAATCACTGGCATCA	ACTGGGCAACTCCAGAAATAAG	98	61098365
DcytB	CATGTGCATTCTCTTCCAAAGTC	CTCCTTGGTGACCGCATTAT	103	20380692
ZnT1	GGTAACAGAGCTGCCTTAACT	GGTAACAGAGCTGCCTTAACT	105	54109718
AP	CGTCAGCCAGTTTGACTATGTA	CTCTCAAAGAAGCTGAGGATGG	138	45382360
SGLT-1	GCATCCTTACTCTGTGGTACTG	TATCCGCACATCACACATCC	106	8346783
SI	CCAGCAATGCCAGCATATTG	CGGTTTCTCCTTACCACTTCTT	95	2246388
18S rRNA	GCAAGACGAACTAAAGCGAAAG	TCGGAACTACGACGGTATCT	100	7262899

^1^ DMT-1, divalent metal transporter-1; DcytB, duodenal cytochrome b; ZnT1, zinc transporter 1; AP, amino peptidase; SGLT-1, sodium-glucose transporter-1; SI, sucrose isomaltase; 18S rRNA, 18S ribosomal RNA subunit.

**Table 3:** Protein and fiber concentrations (g/100 g) of tested peas varieties ^1^.

Variety	Insoluble Fiber	Soluble Fiber	Total Fiber	Total Protein
1-2347-144	22.37 ± 1.26 ^a^	1.31 ± 0.18 ^a^	23.68 ± 1.43 ^a^	22.69 ± 0.06 ^d^
4803-4-78G-L	16.49 ± 1.32 ^c^	0.94 ± 0.39 ^a^	17.43 ± 1.71 ^c^	26.75 ± 0.35 ^a^
4802-8-46Y-L	19.52 ± 1.10 ^abc^	1.08 ± 0.06 ^a^	20.60 ± 1.05 ^abc^	23.22 ± 0.31 ^c^
4802-8-60G-L	20.20 ± 1.87 ^ab^	1.12 ± 0.30 ^a^	21.32 ± 2.17 ^abc^	22.94 ± 0.09 ^cd^
4802-8-87Y-L	17.94 ± 0.12 ^bc^	1.13 ± 0.26 ^a^	19.07 ± 0.14 ^bc^	24.78 ± 0.13 ^b^
CDC Bronco	20.66 ± 1.93 ^ab^	1.30 ± 0.16 ^a^	21.97 ± 1.77 ^ab^	22.50 ± 0.90 ^d^

^1^ Values are means ± standard error of the mean (SEM) (*n* = 3 replicates). ^a–d^ Treatment groups not indicated by the same letter are significantly different (*p* ≤ 0.05).

**Table 4:** Experimental cumulative feed intake ^1^.

	Feed Intake (g)					
Pea Diet	Day 7	Day 14	Day 21	Day 28	Day 35	Day 42
1-2347-144	329.6 ± 26.7 ^a^	699.2 ± 55.4 ^a^	1210.9 ± 120.7 ^a^	1773.1 ± 105.8 ^a^	2511.2 ± 86.4 ^a^	3272.1 ± 115.6 ^a^
4803-4-78G-L	331.1 ± 21.2 ^a^	706.7 ± 50.6 ^a^	1229.6 ± 95.6 ^a^	1579.6 ± 328.0 ^a^	2370.9 ± 348.9 ^a^	3266.8 ± 340.3 ^a^
4802-8-46Y-L	390.8 ± 11.6 ^a^	797.7 ± 58.5 ^a^	1420.9 ± 134.6 ^a^	2051.6 ± 180.1 ^a^	2822.3 ± 230.4 ^a^	3691.3 ± 225.5 ^a^
4802-8-60G-L	351.6 ± 7.8 ^a^	729.8 ± 17.8 ^a^	1283.5 ± 30.3 ^a^	1898.6 ± 5.8 ^a^	2698.8 ± 13.1 ^a^	3644.7 ± 35.7 ^a^
4802-8-87Y-L	370.4 ± 17.1 ^a^	742.0 ± 74.2 ^a^	1312.2± 158.6 ^a^	1934.7 ± 167.2 ^a^	2781.9 ± 182.2 ^a^	3769.6 ± 186.9 ^a^
CDC Bronco	353.3 ± 12.2 ^a^	735.1 ± 28.9 ^a^	1299.4 ± 73.6 ^a^	1901.0 ± 56.6 ^a^	2664.4 ± 49.8 ^a^	3530.4 ± 60.9 ^a^
No pea	224.6 ± 29.1 ^b^	293.9 ± 28.8 ^b^	428.3 ± 48.2 ^b^	609.5 ± 66.3 ^b^	799.5 ± 105.2 ^b^	930.6 ± 133.1 ^b^

^1^ Values are means ± SEM (*n* = 15 animals per treatment group). ^a,b^ Treatment groups not indicated by the same letter are significantly different (*p* ≤ 0.05).

**Table 5:** Experimental cumulative iron intake ^1^.

	Iron Intake (mg)					
Pea Diet	Day 7	Day 14	Day 21	Day 28	Day 35	Day 42
1-2347-144	11.77 ± 0.95 ^b^	24.97 ± 1.98 ^a^	43.25 ± 4.31 ^a^	63.33 ± 3.78 ^a^	89.69 ± 3.09 ^a^	116.87 ± 4.13 ^b^
4803-4-78G-L	13.07 ± 0.84 ^ab^	27.89 ± 2.00 ^a^	48.53 ± 3.78 ^a^	62.35 ± 12.95 ^a^	93.59 ± 13.77 ^a^	128.95 ± 13.43 ^ab^
4802-8-46Y-L	14.07 ± 0.42 ^a^	28.73 ± 2.11 ^a^	51.18 ± 4.85 ^a^	73.89 ± 6.49 ^a^	101.65 ± 8.30 ^a^	132.95 ± 8.13 ^ab^
4802-8-60G-L	12.75 ± 0.28 ^ab^	26.47 ± 0.65 ^a^	46.56 ± 1.10 ^a^	68.87 ± 0.21 ^a^	97.89 ± 0.48 ^a^	132.21 ± 1.29 ^ab^
4802-8-87Y-L	14.11 ± 0.65 ^a^	28.26 ± 2.83 ^a^	49.98 ± 6.04 ^a^	73.69 ± 6.37 ^a^	105.96 ± 6.94 ^a^	143.57 ± 7.12 ^a^
CDC Bronco	13.14 ± 0.46 ^ab^	27.35 ± 1.07 ^a^	48.35 ± 2.74 ^a^	70.73 ± 2.11 ^a^	99.14 ± 1.85 ^a^	131.36 ± 2.27 ^ab^
No pea	6.20 ± 0.80 ^c^	8.11 ± 0.80 ^b^	11.82 ± 1.33 ^b^	16.82 ± 1.83 ^b^	22.07 ± 2.18 ^b^	25.69 ± 3.67 ^c^

^1^ Values are means ± SEM (*n* = 15 animals per treatment group). ^a–c^ Treatment groups not indicated by the same letter are significantly different (*p* ≤ 0.05).

**Table 6:** Experimental body weights ^1^.

	Body Weights (kg)					
Pea Diet	Day 7	Day 14	Day 21	Day 28	Day 35	Day 42
1-2347-144	0.133 ± 0.004 ^a^	0.327 ± 0.010 ^a^	0.547 ± 0.009 ^a^	0.904 ± 0.031 ^b^	1.326 ± 0.082 ^bc^	1.820 ± 0.130 ^b^
4803-4-78G-L	0.137 ± 0.006 ^a^	0.334 ± 0.013 ^a^	0.576 ± 0.022 ^a^	0.997 ± 0.037 ^ab^	1.447 ± 0.058 ^ab^	2.040 ± 0.100 ^ab^
4802-8-46Y-L	0.137 ± 0.011 ^a^	0.337 ± 0.025 ^a^	0.578 ± 0.043 ^a^	0.994 ± 0.067 ^ab^	1.384 ± 0.085 ^bc^	1.880 ± 0.110 ^ab^
4802-8-60G-L	0.136 ± 0.005 ^a^	0.334 ± 0.026 ^a^	0.563 ± 0.044 ^a^	0.974 ± 0.050 ^ab^	1.393 ± 0.076 ^bc^	1.930 ± 0.110 ^ab^
4802-8-87Y-L	0.131 ± 0.006 ^a^	0.322 ± 0.016 ^a^	0.561 ± 0.037 ^a^	1.024 ± 0.049 ^a^	1.536 ± 0.059 ^a^	2.140 ± 0.070 ^a^
CDC Bronco	0.131 ± 0.002 ^a^	0.317 ± 0.006 ^a^	0.541 ± 0.008 ^a^	0.922 ± 0.019 ^b^	1.300 ± 0.006 ^c^	1.840 ± 0.020 ^b^
No pea	0.072 ± 0.003 ^b^	0.090 ± 0.005 ^b^	0.118 ± 0.006 ^b^	0.161 ± 0.009 ^c^	0.201 ± 0.011 ^d^	0.240 ± 0.010 ^c^

^1^ Values are means ± SEM (*n* = 15 animals per treatment group). ^a–d^ Treatment groups not indicated by the same letter are significantly different (*p* ≤ 0.05). Body weights averaged 38 g at the start of the experiment.

**Table 7:** Experimental total body hemoglobin iron (Hb-Fe) ^1^.

	Hb-Fe (mg)			
Pea Diet	Day 7	Day 21	Day 35	Day 42
1-2347-144	4.981 ± 0.152 ^a^	20.845 ± 0.339 ^a^	55.186 ± 3.392 ^bc^	97.790 ± 7.150 ^b^
4803-4-78G-L	5.496 ± 0.245 ^a^	23.452 ± 0.914 ^a^	56.839 ± 2.276 ^ab^	107.280 ± 5.140 ^ab^
4802-8-46Y-L	5.539 ± 0.444 ^a^	22.967 ± 1.727 ^a^	51.886 ± 3.200 ^bc^	96.610 ± 5.600 ^ab^
4802-8-60G-L	5.301 ± 0.207 ^a^	21.814 ± 1.668 ^a^	48.939 ± 2.659 ^bc^	96.980 ± 5.480 ^ab^
4802-8-87Y-L	4.730 ± 0.223 ^a^	19.886 ± 1.314 ^a^	57.088 ± 2.198 ^a^	116.910 ± 3.590 ^a^
CDC Bronco	4.177 ± 0.062 ^a^	17.249 ± 0.268 ^a^	45.414 ± 0.209 ^c^	100.450 ± 1.250 ^b^
No pea	2.354 ± 0.090 ^b^	4.048 ± 0.206 ^b^	6.447 ± 0.348 ^d^	9.480 ± 0.550 ^c^

^1^ Values are means ± SEM (*n* = 15 animals per treatment group). ^a–d^ Treatment groups not indicated by the same letter are significantly different (*p* ≤ 0.05). Total body hemoglobin iron averaged 0.65 milligrams at the start of the experiment.

**Table 8:** Experimental hemoglobin maintenance efficacy (HME) ^1^.

	HME (%)		
Pea Diet	Day 21	Day 35	Day 42
1-2347-144	37.44 ± 3.81 ^a^	56.21 ± 4.66 ^a^	80.58 ± 8.13 ^a^
4803-4-78G-L	39.82 ± 3.85 ^a^	57.37 ± 8.61 ^a^	78.90 ± 7.61 ^a^
4802-8-46Y-L	34.35 ± 2.52 ^ab^	45.72 ± 0.79 ^ab^	70.18 ± 0.23 ^ab^
4802-8-60G-L	37.29 ± 4.09 ^a^	44.56 ± 2.31 ^ab^	71.66 ± 4.45 ^ab^
4802-8-87Y-L	32.22 ± 2.51 ^ab^	49.66 ± 2.29 ^ab^	61.10 ± 1.26 ^b^
CDC Bronco	27.24 ± 1.85 ^b^	41.63 ± 0.96 ^b^	62.92 ± 1.40 ^b^
No pea	14.57 ± 1.33 ^c^	19.04 ± 2.18 ^c^	28.53 ± 3.03 ^c^

^1^ Values are means ± SEM (*n* = 15 animals per treatment group). ^a–c^ Treatment groups not indicated by the same letter are significantly different (*p* ≤ 0.05).

**Table 9:** Hepatic iron and ferritin protein concentrations ^1^.

Pea Diet	Liver Iron (µg/g)	Liver Ferritin (AU)
1-2347-144	96.49 ± 6.52 ^a^	1.078 ± 0.014 ^a^
4803-4-78G-L	73.30 ± 7.58 ^b^	1.084 ± 0.015 ^a^
4802-8-46Y-L	77.61 ± 17.72 ^b^	1.063 ± 0.009 ^a^
4802-8-60G-L	87.46 ± 4.98 ^ab^	1.050 ± 0.005 ^a^
4802-8-87Y-L	71.88 ± 4.79 ^b^	0.469 ± 0.160 ^b^
CDC Bronco	91.34 ± 9.79 ^ab^	0.257 ± 0.017 ^c^
No pea	75.71 ± 6.29 ^b^	0.280 ± 0.007 ^c^

^1^ Values are means ± SEM (*n* = 12 animals per treatment group). ^a–c^ Treatment groups not indicated by the same letter are significantly different (*p* ≤ 0.05). Total iron concentrations were measured as micrograms per gram of liver tissue (wet weight). Liver ferritin concentrations were measured as arbitrary units of liver tissue (wet weight).

**Table 10:** Serum iron concentrations ^1^.

	Serum Iron (µg/µL)			
Pea Diet	Day 7	Day 21	Day 35	Day 42
1-2347-144	2.089 ± 0.161 ^a^	1.682 ± 0.120 ^b^	2.226 ± 0.243 ^ab^	2.116 ± 0.183 ^a^
4803-4-78G-L	1.604 ± 0.108 ^a^	2.322 ± 0.198 ^ab^	2.633 ± 0.451 ^a^	2.104 ± 0.280 ^a^
4802-8-46Y-L	3.029 ± 0.636 ^a^	2.596 ± 0.700 ^ab^	1.795 ± 0.225 ^b^	2.349 ± 0.289 ^a^
4802-8-60G-L	2.383 ± 0.282 ^a^	2.058 ± 0.170 ^b^	1.583 ± 0.106 ^b^	2.240 ± 0.218 ^a^
4802-8-87Y-L	2.767 ± 0.774 ^a^	2.812 ± 0.425 ^a^	1.578 ± 0.144 ^b^	2.132 ± 0.178 ^a^
CDC Bronco	1.936 ± 0.237 ^a^	1.829 ± 0.223 ^b^	1.670 ± 0.190 ^b^	2.292 ± 0.224 ^a^
No pea	2.248 ± 0.490 ^a^	1.526 ± 0.215 ^ab^	1.488 ± 0.088 ^b^	2.105 ± 0.187 ^a^

^1^ Values are means ± SEM (*n* = 12 animals per treatment group). ^a,b^ Treatment groups not indicated by the same letter are significantly different (*p* ≤ 0.05).

**Table 11:** Pectoral muscle glycogen concentrations (AU) ^1^.

Pea Diet	Day 21	Day 42
1-2347-144	0.020 ± 0.012 ^a^	0.044 ± 0.010 ^ab^
4803-4-78G-L	0.023 ± 0.012 ^a^	0.037 ± 0.006 ^b^
4802-8-46Y-L	0.040 ± 0.011 ^a^	0.041 ± 0.026 ^ab^
4802-8-60G-L	0.031 ± 0.008 ^a^	0.055 ± 0.011 ^a^
4802-8-87Y-L	0.024 ± 0.007 ^a^	0.053 ± 0.005 ^a^
CDC Bronco	0.029 ± 0.034 ^a^	0.034 ± 0.003 ^a^
No pea	0.023 ± 0.004 ^a^	0.033 ± 0.008 ^b^

^1^ Values are means ± SEM (*n* = 5 animals per treatment group. ^a,b^ Treatment groups not indicated by the same letter are significantly different (*p* ≤ 0.05).Glycogen concentrations were measured as milligrams per milliliter of pectoral tissue (wet weight).
